# Managerial Discretion, Market Failure and Democracy

**DOI:** 10.1007/s10551-022-05152-8

**Published:** 2022-06-30

**Authors:** Michael Bennett

**Affiliations:** Department of Philosophy, University of Nottingham, University Park, Nottingham, NG7 2RD UK

**Keywords:** Market failures approach, Political Corporate Social Responsibility, Business ethics and democracy

## Abstract

Managers often have discretion in interpreting their ethical requirements, and they should seek democratic guidance in doing so. The undemocratic nature of managerial ethical discretion is shown to be a recurring problem in business ethics. Joseph Heath’s market failures approach (MFA) is introduced as a theory better positioned to deal with this problem than other views. However, due to epistemic uncertainty and conceptual indeterminacy, the MFA is shown to allow a much wider range of managerial discretion than initially appears. The paper explores how this range can be narrowed down with democratic input, comparing models based on formal state institutions and on the informal public sphere. A case study from the pharmaceutical industry illustrates the merits of the informal public sphere approach.

## Introduction

In August 2019, a group of 181 CEOs of the largest US corporations known as the “business roundtable” issued a new “Statement on the Purpose of a Corporation”. According to their press release, the statement “Moves Away from Shareholder Primacy”, to declare:

While each of our individual companies serves its own corporate purpose, we share a fundamental commitment to all of our stakeholders… customers… employees… suppliers… the communities in which we work… shareholders… Each of our stakeholders is essential. We commit to deliver value to all of them (“Business Roundtable Redefines the Purpose of a Corporation to Promote ‘An Economy That Serves All Americans’” 2019).

Among many responses to the statement, an objection from *The Economist* stands out:

It is not clear how CEOs should know what “society” wants from their companies. The chances are that politicians, campaigning groups and the CEOs themselves will decide—and that ordinary people will not have a voice. Over the past 20 years industry and finance have become dominated by large firms, so a small number of unrepresentative business leaders will end up with immense power to set goals for society that range far beyond the immediate interests of their company. (The Economist, 2019)

This is a legitimate concern. The issue of democratic accountability and the discretionary power of managers represents a serious problem for business ethics and political theories of the corporation.

Elsewhere, I have written about this in terms of institutional design and the trade-offs attached to different policies for curbing corporate power [Blinded for reviewing]. Here, my focus is on the question of what we actually want firms to do—how business managers should ideally conduct themselves.

My answer will be framed as a revision to the theory of business ethics I find most compelling, the market failures approach (MFA) articulated by Joseph Heath (2014).^1^ Contrary to appearances, the MFA leaves significant room for disagreement about what managers should do. The resolution of this disagreement solely at the discretion of managers is problematic from the perspective of democracy. Faced with this situation, managers should wherever possible try to defer to democratic processes in interpreting their duties. These democratic processes can be found in the public sphere of civil society as well as in the formal political apparatus.

In making this amendment to the MFA, I aim for a degree of reconciliation between Heath’s more apolitical approach and the rival school of “Political Corporate Social Responsibility” (PCSR). PCSR as articulated by Andreas Scherer and Guido Palazzo (2007; 2011) holds that company policies should be determined by deliberative democratic engagement with stakeholders and the wider public.^2^ My account gives some content to Jeffrey Smith’s notion of business ethics “walking a fine line along the institutional division of labor between market and state” (Smith, 2019, p. 138).

The article is organised in three main parts. Part one sets out the democratic problem of managerial discretion and introduces the market failures approach. Part two then demonstrates how the MFA stumbles on the problem of managerial discretion because it allows for a much wider range of practical disagreement than initially appears. Part three discusses how managerial discretion can be ameliorated with democratic input, comparing models based on formal state institutions and on the informal public sphere. A case study from the pharmaceutical industry helps to illustrate the merits of the informal public sphere approach. Part four compares my argument with related recent work in business ethics.

## The Problem of Managerial Discretion

I begin with the basic problem of managerial discretion. I trace this concern in an underappreciated aspect of Milton Friedman’s famous critique of stakeholder-oriented corporate social responsibility. I then set out how the problem interacts with the two most familiar theories of business ethics, which stand as polar opposites to one another: stakeholder theory and shareholder value maximisation. Finally, I summarise the MFA to set the stage for what follows.

### The Problem

As I will use the term, *managerial discretion* is a feature of a normative theory of business ethics. Managerial discretion arises to the extent that the theory does not provide a manager with unambiguous guidance about what to do.

This definition requires a bit of unpacking. First, this definition is about the room for discretion allowed by a normative theory. It is not about how much empirical room for manoeuvre a manager actually has before someone will stop them or punish them. Second, managerial discretion comes in degrees. Seldom (or perhaps never) is any guidance in life truly unambiguous; yet, we can meaningfully distinguish between guidance which is more or less clear or restrictive.

On this account, discretion arises from two sources, epistemic and conceptual. Epistemic uncertainty creates room for discretion when it is difficult to determine what the theory recommends in a given situation. Epistemic uncertainty often gives rise to reasonable disagreement. Suppose we expect managers to focus single-mindedly on shareholder value maximisation. Even if this goal is clear and determinate in theory, there is usually a great deal of epistemic uncertainty about how best to achieve it. Were it otherwise, managers would be little more than co-ordinators and not especially distinguished members of their firms.

Conceptual indeterminacy arises when the theory does not fully determine what should be done, even if epistemic uncertainty is not an issue (that is, even if we have all the relevant knowledge and ability to process it). Suppose we ask managers not merely to promote shareholder value but to promote the public good. The public good appealed to in such expectations is usually very ill defined. Often, as in the business roundtable statement, invocations of the public good are described in terms of stakeholder interests. Yet this still remains highly unspecified, in at least three ways. First, there is a boundary problem in determining the category of stakeholder. This question appears both in terms of the identification of stakeholder constituencies as such (i.e. are competitor firms stakeholders?) and the ascription of individuals to these constituencies (how far through supply networks should one go in identifying one’s suppliers?). Second, there is a weighing of interests problem between identified stakeholders. When the interests of (e.g.) workers, customers and shareholders conflict, how should a manager weigh these conflicting interests? Third, there is a question about what interests are: how do particular goods such as pleasure, knowledge or freedom contribute to someone’s overall interests?

As long as these variables remain undefined, managers have a wide range of discretion about how to interpret their ethical obligations. Such discretion is problematic from the perspective of basic democratic norms. Decisions by business managers are often highly consequential, seriously affecting lives of large groups of people. These decisions are also morally charged, involving trade-offs between competing values. In a democratic society, we normally assume that consequential, value-laden choices require some kind of democratic process. Changing the law, for example, has wide-ranging consequences and important moral dimensions. Accordingly, constitutional structures exist to make sure legislation tracks democratic processes, particularly through competitive elections for legislative assemblies.

There are many possible normative foundations for this basic democratic norm. Democratic procedures might be favoured for reasons of procedural fairness and respecting citizens as equals (e.g. Christiano, 2010b; Rawls, 1996; Waldron, 1999). Democracy might be favoured as a way of enhancing the epistemic quality of the decisions (e.g. Estlund, 2009; Goodin & Spiekermann, 2018; Landemore, 2013). Here, I will attempt to stay agnostic on how the basic democratic norm is grounded, and simply take it as a premise for what follows.

The problem of managerial discretion is that the public good is radically unspecified, and so asking managers to pursue the public good gives them a wide range of discretion. However, this discretion seems unjustified given our normal commitment to deciding consequential moral choices of this nature democratically.

### Milton Friedman

A similar argument can be found in Milton Friedman’s seminal critique of corporate social responsibility. Today, this text is mainly remembered as a principal-agent story: managers have a duty to obey the wishes of their shareholders. However, the argument that actually takes up more space in the text is a democratic one. Friedman argues that a manager who sincerely and diligently pursues a broader social purpose.

…becomes in effect a public employee, a civil servant, even though he remains in name an employee of a private enterprise. On grounds of political principle, it is intolerable that such civil servants–insofar as their actions in the name of social responsibility are real and not just window-dressing–should be selected as they are now. If they are to be civil servants, then they must be elected through a political process (Friedman, 1970).

In context, it is clear that Friedman put this argument forward as part of a reductio: corporate social responsibility makes business managers into public servants; public servants should be elected; this would be socialism; socialism is bad; therefore, corporate social responsibility is bad. Nonetheless, the argument raises a valid concern.

We can think of treating managers like public officials as part of a *governmental model* for getting managerial decisions to track the interests of the public. Friedman argues for an alternative *market model*, where managerial decisions are directed towards the public interest by the pressure of competition [blinded for reviewing]. This model operates indirectly: the idea is that the pursuit of profit, under conditions of competition, leads in the aggregate (as if by an invisible hand) to the general interest. On this model, managers should pursue shareholder value, not pursue the public good directly.

The problem of managerial discretion, thus, arises because the demand that managers pursue the public good directly sits ill with the market model. The market model is supposed to promote the public good indirectly, and there is no reason to think that market signals will facilitate managers pursuing it directly. The problem of discretion, thus, arises to the extent that we move away from an indirect market mechanism and towards asking managers to directly pursue the public good in the manner of public officials. Once we take this step towards a more governmental model, the lack of an accompanying democratic process appears as a problem.

Friedman’s own solution, of course, was to avoid taking that step. He advocated a depoliticised ethic of shareholder value maximisation that delivers clear and determinate recommendations with little room for discretion. However, this version of business ethics has other disadvantages. Although I do not have room to substantiate the objection here, I share the common concern that shareholder value maximisation is insufficiently morally demanding. I will, therefore, proceed directly to the theory of business ethics which I find most compelling, and which attempts to deal without the problem of managerial discretion without blunting business ethics’ critical edge.

### The Market Failures Approach to Business Ethics

This subsection introduces the market failures approach (MFA) to business ethics, particularly as advanced by Joseph Heath. Since this mainly consists of exposition, those already familiar with the approach may wish to skip ahead to the next section.

The approach starts from a conventional or instrumental understanding of business law and ethics: the market is an artificial institution which exists to promote certain human ends. For Heath, the implicit morality of the market system is economic efficiency. The first fundamental theorem of welfare economics shows that, within the framework of perfect competition, self-interest on the part of businesses and other economic actors leads to pareto-optimality. However, the requirements of perfect competition in this sense are very stringent. Where there are “market failures” (where these conditions do not obtain), self-interest by businesses does not necessarily lead to economic efficiency.

Heath’s suggestion for business ethics is that managers should behave *as if* they operated in a perfectly competitive market. In other words, they should refrain from taking advantage of market failures—they should not pollute, or deceive their customers—which would not be possible in a perfectly competitive market. Businesses should only partake in transactions which contribute to efficiency. They should avoid transactions which benefit the business only at the expense of contractual partners or bystanders.

State regulation can also prevent firms from exploiting market failures—pollution or deception could simply be outlawed. However, it will always be more efficient if managers voluntarily refrain from exploiting market failures, rather than being forced not to by the state. If managers treat law and punishment simply as additional variables in their profit-maximisation calculus, monitoring and enforcement costs will be vastly increased. The situation for business ethics in this regard is not so different to that of interpersonal ethics. In ordinary interpersonal conduct, we take it for granted that law and punishment should be viewed as a backstop to catch egregious failures of individual morality, and not simply as the “rules of the game” within which all moves are acceptable.

Like shareholder value maximisation, the MFA avoids giving managers broad discretion by making asking them to pursue the public good directly; instead, managers have the telic goal of maximising shareholder value. However, Heath’s solution puts limits on the pursuit of shareholder value in a much more convincing way than Friedman’s. To some extent, Friedman deals with cases of unethical business behaviour by asking managers to constrain their profit seeking by conformity to the rules “embodied in ethical custom” (Friedman, 1970). But there is no attempt to justify or explain this, and it seems unsatisfyingly ad-hoc. By contrast, Heath justifies a detailed list of prohibitions by referring to the economic preconditions for markets to fulfil their social role of promoting efficiency.

For these reasons, the MFA seems in a good position to deal with the problem of managerial discretion. Indeed, I believe it does advance on previous theories in this respect. However, it is also less successful than it initially appears. In the next section, I argue that the MFA delivers much less determinate recommendations than one might think, leaving space for the politics of managerial discretion to raise its head once again.

## The Persistence of Discretion in the MFA

The MFA fails to deliver determinate recommendations for two reasons. The first issue is epistemic uncertainty, relating to the complications arising from the theory of the second best. The second is conceptual indeterminacy, relating to the nature of the Pareto criterion.

### Epistemic Uncertainty

Even when a goal is theoretically well defined, knowing what to do to meet that goal can be extremely difficult.

This can arise for the MFA due to what is known as the “problem of the second best” (Lipsey & Lancaster, 1956). To recall, the fundamental theorem of welfare economics shows that a pareto-optimal equilibrium arises under certain specified conditions, such as absence of externalities and absence of market power. The problem of the second best is so called because it arises once we start asking what we should do if the best option (full conditions of perfect competition leading to pareto-optimality) is unobtainable. The problem is that under these “second-best” circumstances, the fundamental theorem ceases to offer any guidance.

The natural temptation is to think that fewer departures from perfect competition lead to a more efficient outcomes than more departures from perfect competition. But this cannot be relied upon. As soon as there are departures from perfect competition along more than one dimension, we cannot be sure that restoring perfect competition in one dimension but not the other will make things better off. The basic reason is that without additional information, we do not know whether the two different departures are compounding the inefficiency or cancelling each other out. The problem of the second best is normally understood as a problem for policy makers seeking to advance efficiency. However, an example will show how the efficiency focus of the MFA transfers a version of this problem onto business managers.

Consider oil.^3^ Standard Oil was the original archetype of an anti-competitive monopoly, and its break-up was the defining moment in the arrival of serious antitrust law in the USA. Even afterwards, Standard Oil’s five main successors along with Royal Dutch Shell and British Petroleum dominated the global market in the mid-twentieth century, becoming known as the “Seven Sisters”. Since the 1970s, market power has shifted to perhaps the most famous cartel of all, the Organization of the Petroleum Exporting Countries (OPEC). Unusually, OPEC explicitly operates as a cartel, protected from antitrust actions but the fact that its members are sovereign states. It is safe to say that oil has a long history of industrial concentration and anti-competitive practices. Oil producers have used their market power to raise prices and reap super-normal profits in equilibrium. This is a market failure; in a perfectly competitive market there would be too many competitors for any one producer to influence the price, and so prices would be lower. The market failure approach would, thus, recommend that oil producers avoid taking advantage of their market power by keeping prices lower than it could get away with.

However, the oil industry also lies at the heart of an even more important market failure—indeed, the greatest market failure of all. As the leading cause of anthropogenic global climate change, the burning of oil (alongside other fossil fuels) constitutes a massive negative externality. Oil users impose massive costs on others which are not compensated for. This is reflected in a price of oil that is inefficiently low, and thus, a quantity of consumption that is inefficiently high.

If either market failure was the only market imperfection at issue, the MFA would give clear guidance. But when both market failures are combined, it’s much harder to know what to do. The two market failures in this case have some tendency to cancel each other out. Ordinarily, higher prices due to monopoly power would be a bad thing. But in this case, higher prices have helped to offset the inefficiently low prices as a result of negative externalities. At the same time, while these market failures are offsetting, we cannot be sure exactly where the balance lies. To be clear, I am not suggesting that oil producers were thinking about climate change when they formed a cartel to fix prices. It’s just that if producers had abjured cartelisation in an attempt to behave more ethically, the end result would probably have been worse.

The possibility of cases like this means that once we have departures from perfect competition along multiple dimensions, working out what we should do to enhance efficiency becomes epistemically much more difficult. The conditions of perfect competition no longer offer much guidance.

Critics such as Jeffrey Moriarty (2019; see also Steinberg, 2017) have recognised the problem which the second best presents for the MFA, and Heath has responded:

Medical students, while being trained to diagnose illness, are often reminded that “common things are common”, and that “when you hear hoofbeats, think horses, not zebras”. This is because, having memorized the symptoms of various exotic diseases, they have a tendency to overdiagnose them, forgetting that almost every patient who presents with flu symptoms has the flu, most people with headaches just have headaches, and so on. By focusing so much on the second-best theorem, Moriarty is basically getting distracted by a zebra. When firms produce pollution, it’s almost always bad. When firms collude to fix prices, it’s almost always bad (Heath, 2019, p. 25).

There is much to sympathise with in this response. While the second-best theorem shows that there is no conceptual guarantee that reducing a particular market imperfection will improve efficiency, it is still very likely in many cases.

Nonetheless, I think Heath is overly sanguine about the likelihood of offsetting imperfections. The example of the oil industry shows that this is no mere academic exercise. Indeed, the example features a clash between the very two imperfections Heath mentions in his response: externalities and price fixing. More generally, even if the MFA provides the best systematisation of our judgements about relatively uncontroversial cases, it is reasonable for us to expect a theory of business ethics to say something about more difficult cases.

To be clear, the problem of the second best does not invalidate the whole idea of an efficiency-based or Paretian business ethics. It is still coherent to think of the function of business ethics as being to rule out business strategies that lead to pareto-suboptimal outcomes. The problem is just that it becomes much harder to know what that actually implies in practice once we cannot fully rely on the conditions for perfect competition as a cheat sheet. That is why this is an epistemic problem. (Amit Ron and Abraham Singer (2020) include this in what they call “the problem of judgement”). Deciding which business strategies are conducive to efficiency can be difficult, and require a careful examination of the particular context. This difficulty means that there can be considerable room for disagreement. And this room for disagreement re-opens the scope for managers’ discretion.^4^

### Conceptual Indeterminacy

The epistemic uncertainty problem is that we do not know how to get to attain the pareto-optimum. The conceptual indeterminacy problem is that even if we know how to attain an optima, there are multiple optima and we also need to choose between them.

The MFA’s focus on Pareto-optimality builds in a degree of conceptual indeterminacy from the beginning. The Pareto criterion recommends states of affairs where someone can be better satisfied without diminishing the satisfaction of anyone else. However, the criterion gives no guidance in cases where someone can be better satisfied at the expense of someone else. It has nothing to say about distributive questions. The choice between Anne being rich while Bob is poor and Bob being rich while Anne is poor is a choice between two pareto-optima. Such questions are simply undecidable using the Pareto criterion alone.

This much is familiar. However, what often goes underappreciated that Pareto-undecidable distributive choices frequently arise in the context of institutions designed to enhance efficiency and not merely in the explicitly distributive contexts of “initial endowments” and taxation. This is the central lesson of Heath’s article “The Benefits of Co-operation” (2006). According to Heath, there are five qualitatively different mechanisms of co-operation which enable efficiency gains, and these mechanisms often come into conflict with one another.

The MFA is rooted in the mechanism of co-operation Heath calls *gains from trade*, since that is the mechanism at work in the fundamental theorem of welfare economics. Gains from trade are possible when two people prefer what each other has more than what they begin with. By exchanging, they will each be better off, without adding any new “stuff” to the economy. Gains from trade are maximised when the market clears and every last Pareto-improving exchange is undertaken.

Both imperfections in the previous example concerned gains from trade. By exercising market power, oil producers raise prices above the market-clearing rate, leaving a residue of potentially efficiency-enhancing trades that are not undertaken because producers prefer the additional profit from keeping prices high for their other customers. Negative externalities such as pollution can also be understood as harming gains from trade, or rather as a set of unwanted and inefficient “trades”. The problem with pollution, from this perspective, is that polluters do not have to negotiate a price for their pollution with those whom they affect. Instead, they pollute for free, and so the quantity of pollution is inefficiently high (Heath, 2006, 332; Coase, 1960). Looking upstream to the oil market, this leads the quantity of oil being traded to be inefficiently large, a problem that would be remedied by higher prices.

Consider now an example of a conflict between two different mechanisms of co-operation. The black liquid in this case is not oil, but cola. Suppose a fizzy drinks producer has market power which enables them to raise prices above the market-clearing rate. As with the oil producers, this is a straightforward market failure.

But suppose now that many of the customers of this company are worse-off from consuming these fizzy drinks. They experience what Heath calls *dynamic preference inconsistency* due to discounting future experiences:

For example, given a choice between a check for $100 that can be cashed right away and a check for $200 that can be cashed in three years, many people will choose the former. Many of these same people, however, when given a choice between a $100 check that can be cashed in six years and a $200 check that can be cashed in nine years will take the $200 check. This preference structure will generate dynamic inconsistency: the individual who would take the $100 check right away and chooses the $200 check that can only be cashed in nine years will want to change the latter decision in six years’ time (Heath, 2006*, 325).*

Suppose preferences for fizzy drinks are like this: in the long run, some people would choose a low level of consumption for the sake of health benefits; however, in the very short run, they discount the future and focus on the refreshing beverage before them.^5^ The result is never attaining the desired long-run (low) level of consumption and experiencing continual regret. Philosophers may describe this behaviour in various ways such as weakness of will or a failure of autonomy, but for our purposes the important thing is that it gives rise to a market failure. In a perfectly competitive market, consumers are assumed to have stable preferences which rule out these kinds of cycles of regret. Because of these dynamically inconsistent preferences, an inefficiently large quantity of fizzy drinks is being consumed.

People respond to dynamic preference inconsistencies through a mechanism of co-operation Heath refers to as *self-binding*. When we are aware that our long-term ambitions are repeatedly frustrated by our short-term desires, we can take steps to reconfigure our environment to prevent ourselves from acting on those desires. This becomes a mechanism of social co-operation when we enlist others to help us in these self-binding strategies (e.g. asking your employer for higher pension contributions rather than a higher salary).

As with the oil example, the MFA is unable to give clear advice because market imperfections pull in opposite directions. If the fizzy drinks company chooses high prices, then it will reduce efficiency by reducing gains from trade. On the other hand, if the company chooses low prices, then it would reduce efficiency by making it harder for consumers with dynamic preference inconsistencies to self-bind. As before, this might be interpreted as an instance of the problem of the second best: where there are multiple types of co-operation implicated, and not merely gains from trade, we cannot rely on the conditions of perfect competition as guidance for achieving efficiency.

However, Heath’s earlier article makes a deeper point. The conflict between mechanisms of co-operation is not merely a matter of uncertainty about which is more important, but a conflict between people who are differently benefited by different mechanisms. To return to our example, if all consumers were equally subject to dynamic preference inconsistencies, then this would indeed be a purely epistemic problem of selecting the price level which would best balance self-binding and gains from trade. However, in a world where this is more of an issue for some consumers than others, this is no longer the case. Consumers for whom the dynamic preference inconsistency is a major problem would prefer higher prices that would have the effect of binding them to consume less fizzy drinks. But other consumers who are perfectly happy with their fizzy drinks consumption would simply prefer lower prices. This leads to a situation with multiple pareto-optima. The choice for the drinks company boss is, thus, not (only) one of figuring out which price level leads to a pareto-optimum. They are also choosing between pareto-optima: choosing which set of customers whose interests will have priority.

Heath wants to stress that efficiency is not the neutral, apolitical concept it can appear to be. As I have put it elsewhere, even if we focus on efficiency, we have a choice of efficiencies to make [blinded for reviewing]. Heath’s focus in the “The Benefits of Co-operation” is solely on this conflict at the level of institutions. His point is directed towards policy makers, as they consider topics such as how vigorously to enforce competition/antitrust, or whether to implement a sugar tax. However, our example shows that not only society-wide institutions but also courses of action by individual businesses can facilitate one mechanism and set back another. While Heath is perceptive about the politics of efficiency in public policy, he does not see that an analogous problem arises for firms, and that this causes problems for his efficiency-focused business ethics.

At root, the indeterminacy problem arises because of the constitutive limits of the Pareto-optimality concept. Resolving the indeterminacy problem (choosing an optima), thus, requires a thicker moral concept(s) than mere Pareto-optimality, in order to distinguish between more or less desirable Pareto-optima. For example, the Kaldor-Hicks principle would recommend the optima with the greatest aggregate net utility. Alternatively, the Rawlsian difference principle would recommend the optima in which the worst-off people are in the best situation. While others have argued that the Paretian focus of the MFA should be supplemented by additional values (Cohen & Peterson, 2019), I have shown that additional values can actually be required in order to make determinate recommendations. The choice between different principles of distributive justice is a paradigm case for democratic decision. However, what I have shown is that this choice of distributive principle remains in the discretion of managers, a clear case of problematic managerial discretion.

In summary, there remains significant room for disagreement about how to interpret the demands of the MFA. This room for disagreement comes from two sources: epistemic uncertainty and conceptual indeterminacy. In both cases, the room for disagreement within the MFA arises in situations where there are departures from the conditions of perfect competition in more than one dimension. These situations are common enough to require a serious answer. Given multiple departures, it becomes difficult to know how to attain a Pareto-optimum, because market failures may either exacerbate or counteract each other; when they counteract, it is likely to be unclear whether they do so partially or fully. This is the epistemic uncertainty. Moreover, what appear as “market failures” from the gains-from-trade perspective of welfare economics can also be characterised as (malfunctioning) alternative mechanisms of co-operation. Different ways of advancing efficiency often conflict with one another, necessitating a choice between different efficiencies. These competing Pareto-optima differentially benefit different parties, and so the choice between them is a matter of distributive justice. This is the conceptual indeterminacy. Together, these sources of disagreement mean the MFA’s guidance to managers is much less clear than it initially appears. Managers retain a large scope of discretion in interpreting their ethical obligations. In the following section, I will look at how this margin of discretion can be reduced.

## Democratising Managerial Discretion

The problem we are left with is that choices about how to interpret the MFA are highly consequential and ethically loaded, and leaving these choices to the judgement of managers is unappealing. There is no particular reason to think that managers are the best people to make these decisions. Normally, we expect ethical choices of this kind to be the outcome of a democratic process. However, simply transforming managers into public officials on the standard governmental model would defeat the purpose of the MFA, which is supposed to be an ethic for competitive enterprise.

There are no entirely satisfying options. However, there are ways that managers can draw on democratic processes to narrow down the scope of their discretion in interpreting the MFA, without thereby negating the competitive nature of business. This discretion cannot be eliminated, but it can be ameliorated. This section sketches some basic models for getting democratic input and maps out the considerations involved in the choice between them.

The first model is for managers to seek ethical guidance from the outputs of formal state institutions, “the spirit of the law”. The second model is to look to debates in the public sphere of civil society for guidance, “the spirit of public opinion”. The third model is to rely on institutionalised stakeholder forums. The third model is really more of a broad category of options, including industry organisations, multi-stakeholder initiatives, quasi-non-governmental organisations and worker representation on boards of directors.

I focus my comparison on the first two models rather than the third category. What makes the formal state and the public sphere models similar to each other and different to the stakeholder forum model is their relation to questions of feasibility. On the one hand, the first and second models are utopian and unconcerned with feasibility in the sense that no strong mechanism is envisioned to force managers to actually take up the guidance on offer. This is in contrast to institutionalised stakeholder forums, which offer at least the possibility of stronger incentives for managers, although this remains difficult in practice.^6^ On the other hand, the formal state and public sphere models are more realistic and concerned with feasibility in the sense that they require no new institution-building or political reform but can be implemented by managers here and now. This contrasts with institutionalised stakeholder forums, the construction of which requires political struggle and administrative work (all the more so if they are supposed to offer strong incentives).

Both sets of questions (how managers should behave here and now and how ethical managerial incentives could be better institutionalised) are legitimate and important. From the external perspective of activists, it would be unfortunate if either side of this contrast were neglected. It is important for civil society groups to attempt to hold managers accountable for their behaviour here and now, and it is also important to try and construct institutions which will lead to better behaviour in the future. Moreover, while there is some trade-off in terms of where activist energy should be spent, there are also important complementarities. In particular, critiquing concrete contemporary corporate activity can help build support for and define desirable features of future institutional change.

In other work, I have examined options in the broad category of institutionalised stakeholder forums, asking how managers might be given meaningful incentives to behave more ethically, and what the costs of such attempts might be. [blinded for reviewing]. Here, I will set aside this category and concentrate instead on the choice between the formal state and public sphere models. This follows the focus in this article on what mangers should do rather than how other parties in society should hold mangers accountable.

### Considerations at Stake

There are many different ways of institutionalising democracy, and different approaches will be preferable in different contexts. Here I follow in particular the suggestion of “deliberative systems” theorists that we should think of deliberative democracy as something that happens in a dispersed way throughout various institutions in society, rather than requiring everyone to come together in one big impossible meeting (Owen & Smith, 2015; Parkinson & Mansbridge, 2013).

It is quite possible for managers to get ethical guidance from both formal state institutions and the public sphere of civil society. However, there is a genuine choice about which side to emphasise and pay more attention to in each case, and a comparison can help illuminate the strengths and weaknesses of each.

I identify three considerations that are at stake, which I refer to as clarity, expertise and inclusivity. As I will show, to the extent that either model performs poorly with regard to each of these considerations, this re-opens the space for managerial discretion and so reduces the utility of the approach.

*Clarity* refers directly to how easily we can get usable guidance which actually narrows down the scope of managerial discretion. This in turn breaks down into two further aspects: unity and granularity.

*Unity* is the advantage of formal state institutions. Constitutional procedures are designed to fix on outcomes with a single authoritative voice, and these outcomes stand as the law of the land or the policy of the government. The debate in the public sphere, on the other hand, never concludes with a vote approving a final text. The debate never stops, and the most a sincere observer can come away with is a more or less vague “sense of the meeting”. The fact that the outputs of the public sphere are not unified makes it harder for managers to draw determinate ethical guidance from them. Insofar as there is disagreement, managers are forced to make an interpretive choice about where the central tendency of public opinion lies. This necessity of interpretation leaves open space for managerial discretion.

*Granularity*, on the other hand, is where the advantage lies with the public sphere. Public debates regularly develop around granular questions of business ethics, often specifically on the practices of individual firms. By contrast, the state may speak with one voice, but it is silent on many particular ethical questions businesses face. Formal political institutions in liberal democracies have very specific functions: legislatures legislate, judiciaries interpret laws in contested cases, and executives manage the government bureaucracy. This functional specificity makes it perilous to try and look at these processes to attempt to answer other questions. Legislators are working towards writing legislation, not issuing general guidelines for dealing with ethical questions. In particular, absence of legislation regulating some type of conduct cannot be taken as evidence that legislators regard that conduct as unproblematic. It could very easily have been the case that legislators would welcome voluntary restraint by companies, but decided that legislation would be counter-productive (perhaps because of expected resistance). The further away the question addressed by legislators lies from the question faced by a manager, the more interpretative work is necessary to extract guidance relevant to the question in hand. Again, this reduces the utility of the approach in reducing managerial discretion.

*Expertise* becomes a consideration insofar as understanding managers’ ethical questions necessitates technical knowledge. Expertise is often assumed to tilt in favour of elitism, but others have argued that inclusive democratic processes tend to process relevant knowledge better than small elite groups (Goodin & Spiekermann, 2018; Landemore, 2013). Managers cannot be expected to directly take on board advice which is based on ignorance of crucial details. Instead, they need to think about the extent to which technical knowledge is required, the extent to which it is lacking in the public sphere or formal institutions and finally to reconstruct how these debates or outcomes might have looked if the requisite technical knowledge had been present. All of these are interpretive questions, and so once again expertise (when required and lacking) becomes a source of continued managerial discretion.

In terms of the contrast between formal state institutions and the informal public sphere, expertise is relevant primarily because there are reasons to think it follows to some extent from granularity, which would make it a point in favour of the informal model. The core political institutions are massively overburdened with the range of topics they have to deal with. Already, it is not realistic to expect legislators to be personally knowledgeable about all of the policies they legislate on. Legislators are highly reliant on their staff of researchers, on their parties and their research staff and in turn on their associated think tanks and research institutes. Moreover, contemporary legislation very often leaves many particulars unspecified and delegates further specification to bureaucratic agencies.^7^ Such delegation is necessary because the legislature simply has too much business to get through to be able to carry their work through to all the details. Another potential advantage of the informal public sphere is that it is possible to envisage a kind of “feedback loop” in which managers’ interpretations of the state of public opinion are themselves open to challenge by civil society groups (Chambers, 2017).

*Inclusivity*, finally, refers to an egalitarian democratic quality. A lack of inclusivity was the complaint against managerial discretion which prompted this entire inquiry. However, the supposedly democratic sources from which we encourage managers to seek guidance can themselves lack inclusivity. This is clearer in formal state institutions, where we can identify undemocratic organs of state (such as the House of Lords in the United Kingdom), or unequal distributions of voting power (such as the geographical bias of the Senate in the USA). The ideal of an egalitarian public sphere is harder to pin down, and judgements of inclusivity are fuzzier.^8^ What seems essential, however, is an ideal of individuals and associations freely engaging one another in rational persuasion, Jurgen Habermas’ “forceless force of the better argument”. Real public spheres fail to meet this ideal in various ways. Rational persuasion is distorted by power relations including gender, class, race and simply by the unequal amounts of money devoted to advocating for different perspectives (Bennett, 2020; Christiano, 2012; Sanders, 1997). State institutions of democracy are themselves dependent on the public sphere and so also inherit inequalities found there.

Uncritically following an undemocratic public sphere or state apparatus would not improve the democratic credentials of managerial decisions. Managers should accordingly be sensitive to these distortions and weigh or filter what they hear based on the inclusivity of the deliberative process. Again, this interpretative exercise re-opens space for managerial discretion. This interpretative discretion in the name of inclusivity is self-limiting, since managerial discretion is itself highly exclusive. Nonetheless, it can reduce the utility of the approach.

### Spirit of the Law, or of Public Opinion?

What I provide here is only a sketch of how to think about the choice between different sources managers can appeal to in narrowing down the range of their discretion, not a comprehensive comparative assessment. A one-size-fits-all answer will not be forthcoming in any case, since the balance of considerations will differ considerably by sector or issue. However, I venture the following conclusion: managers should take guidance from the outcomes of the political process where possible. However, this is unlikely to take us very far, and so ethical managers should focus more on engaging with civil society.

Incorporating democratic input from formal state institutions gives an added impetus to the need to respect the spirit as well as the letter of the law. This is a message that Heath already stresses in the MFA, in the context of many regulations that are designed to remedy market failures. Being a good sportsperson is following the spirit of the game, not just treating the umpires as part of the game that you can manipulate. My contribution here is to point out that this acquires additional force once we see the multiplicity of different ways efficiency can be promoted. Managers should go along with attempts at (e.g.) reducing obesity, even if they could make an efficiency case for doing otherwise, because this is the form of efficiency that a democratic process has decided to prioritise. In many cases, businesses should not be attempting to solve social problems themselves but assisting governments in their attempts to do so.

However, managers face many ethical issues in interpreting the MFA which state laws and policies do not address in any detail. Trying to extend the spirit of the laws to cover such cases requires so much interpretation that this approach ceases to offer much help in reducing managerial discretion. As a result, I suggest that the most important source of guidance for narrowing down managerial discretion in interpreting the MFA is not the formal state apparatus but the informal public sphere.

The contestation of discourses in the media, think tanks, academia and in everyday talk plays a crucial role in democratic deliberation.^9^ Here too a need for interpretation arises, this time not as a result of a potential lack of granularity, but due to a potential lack of unity. Whereas the challenge previously was to identify the spirit of the law, now the challenge is to identity the spirit of public opinion. This need not be a significant challenge in every case. Often, public debate about corporate conduct is structured around companies versus a relatively united opposition of NGOs and campaigners. When this is the case, the imperative for managers is to listen to what critics are saying and try to accommodate their concerns as much as possible within a market failures approach to business ethics. However, on other occasions, businesses may find themselves genuinely caught in the middle of a public sphere pulling them in multiple directions. When the public sphere is genuinely split managers must necessarily fall back on their own judgement. Even here, however, turning to civil society is still helpful for interpreting the demands of the MFA. Even when the public debate does not identify to a clear answer, it will at least narrow down the range of options to a more acceptable menu for bosses to pick from.

We should also remember that a lot of apparent ambiguity in the public sphere surrounding businesses is actually generated by businesses themselves. With fossil fuel companies and climate change, insurance companies and the US healthcare system, tobacco companies and lung cancer, and other examples too numerous to mention, businesses have spent money cultivating “astroturf” civil society groups to support them against grassroots civil society opposition. In reality, not only do many business managers ignore public debate, they actively seek to undermine it. Claims from business managers that opposition to them is not representative of the whole public sphere should, thus, be taken with healthy scepticism. As a corollary, if businesses are going to get useful guidance out of the public conversation, at the very least they need avoid actively spoiling the public conversation.

One might worry that the public sphere is in decline, increasingly fragmented into polarised online echo chambers and awash with misinformation. While increased polarisation is certainly a real phenomena in some countries, I think there is a tendency to exaggerate these kinds of worries (for careful examinations of the topic see the essays in Edenberg & Hannon, 2021; Hannon & Ridder 2021).^10^ In any case, there are reasons to doubt such pathologies will seriously hinder business leaders’ abilities to accurately assess public opinion. The consensus view in the literature on political irrationality is that insofar as ordinary citizens process political information in a biased way, the primary reason is that doing so is cheap for them becasue their political beliefs have little individual impact (Caplan, 2011; Huemer, 2016; Somin, 2013). Business leaders, on the other hand, have a strong interest in accurately understanding the state of public opinion in relation to their companies, and they have public relations staff to assist them in doing so. Above all, I do not envisage managers as sitting back and observing debates in the public sphere; as the following example will indicate, on serious matters I expect the public sphere will come to them.

### Public Sphere Engagement and the Pneumonia Vaccine

To make the idea of engagement in the public sphere more concrete and meaningful, this subsection briefly discusses a real example. My case comes from a campaign on vaccine pricing run by the global non-governmental organisation Medicins Sans Frontiers (MSF) which was directed at the pharmaceutical companies GlaxoSmithKline and Pfizer.

The first thing to point out about the pharmaceutical industry is that it operates a long way from the conditions of perfect competition. By its nature, the industry is heavily reliant on intellectual property, whereas freely available information is a crucial part of the first fundamental theorem of welfare economics. Intellectual property works to create micro-monopolies around each patent. Moreover, at a macro-level, the pharmaceutical industry as a whole is highly concentrated around a few multinationals, including Pfizer and GlaxoSmithKline.

Despite this distance from the conditions of perfect competition, the MFA can still give clear guidance on some ethical issues that arise for pharmaceuticals. Think for example of the aggressive marketing of opioid painkillers. In terms of the MFA, this aggressive marketing clearly exploited market failures. It exploited asymmetries of information between drug companies and patients (and perhaps between companies and doctors). It also exploited dynamic preference inconsistency on the part of patients addicted to the drugs.

However, on other issues, the fact that the industry operates so far from the conditions of perfect competition does make it difficult to use those conditions for ethical guidance. Consider the massive rents that companies get out of products like vaccines. The justification for the profit companies make from these products is supposed to be that they reward and so incentivise research (including all the research that never ends up going anywhere). However, the scale of these profits, particularly in areas like vaccines, is way out of proportion to the most generous estimates of how much drug companies have been spending on research (see e.g. Light et al., 2009).

At this point, one could of course say that the problem lies with state regulation: with the design of intellectual property, and with the way private companies take advantage of publicly funded research in universities. To some extent, this system has been created and sustained by the lobbying of pharmaceutical companies, which is itself a clear breach of the MFA.

Nonetheless, this does not answer the question of how managers of pharmaceutical companies should behave now. Until the system is changed (if ever), how should companies respond? Several options are open, and the MFA does not seem to help us distinguish between them. On the one hand, perhaps drug companies should channel more of their super-normal profits back into research, given that research is the stated justification for the intellectual property system their profits are based on. Alternatively, perhaps the companies should channel their windfalls into lower prices for their customers. But if so, which customers? After all, given their market power, the drug companies have considerable capacity to price discriminate.

My suggestion is that ethically concerned mangers should help answer these questions by engaging with civil society campaigns as manifestations of a deliberative democratic system. In 2015, MSF launched the “Fair Shot Campaign” to campaign for lower prices and improved access to the pneumococcal conjugate vaccine (PCV) (“A Fair Shot” 2019). Pfizer and GlaxoSmithKline were the only producers, and with no generic competitor version on the horizon, there was no market pressure on either company to reduce the cost of their vaccine (ShareAction & Médecins Sans Frontières, 2016). At the time, pneumonia was the single largest cause of childhood deaths for children under five years of age. But many low- and middle-income countries could not afford the vaccine, and MSF itself could also not afford to use it in humanitarian work.

A public–private partnership styled “Gavi, the vaccine alliance” was in place for companies to offer a lower price for vaccines for low-income countries.^11^ However, countries that qualified as middle-income lost this protected status and were exposed to the full profit-maximising behaviour of the drug companies. A country like Jordan was too wealthy to classify for GAVI support but too poor to afford to vaccinate all children of the appropriate age. Jordan carried a particularly heavy burden since the country was resolved to include in its vaccination programme all children among the 2 million Syrian refugees who fled to the country (which has a total population of only 10 million). MSF argued that the lower prices should be extended to humanitarian organisations and to countries like Jordan which were experiencing humanitarian emergencies.

After a period of campaigning and activism followed by direct negotiations, the campaign did have some success. GSK and Pfizer agreed to the creation of a “Humanitarian Mechanism” whereby non-governmental organisations, regardless of where they are working, can purchase PCV at the lowest global price.

In making the case for lower prices for vaccines, MSF had certain democratic credentials. As an organisation, MSF matches the idea of a “civil society organisation” pretty closely. MSF is funded by a large number of small donors motivated by particular moral and political concerns. Unsurprisingly, this donor base is concentrated in richer countries. However, the staff is broadly reflective of the countries the organisation operates in, and the organisation’s campaigning agenda is shaped by its staff. MSF is one of the closest things to a global civil society organisation that exists, which is appropriate given the global nature of the topic. In any case, MSF did not simply demand lower prices based on its own legitimacy, but made an argument for vaccine prices in the public sphere. Their intention was to embarrass the drug companies before public opinion: globally, and especially in those countries where the companies were most sensitive.

The campaign also sought to move beyond the informal public sphere to gain traction with formal state institutions or their supranational equivalents. In 2015, the World Health Assembly (WHA) (the legislative body of the World Health Organisation (WHO)) passed a motion recapitulating the campaign’s themes (World Health Assembly, 2015; For reporting see Cooper, 2016). The resolution shows how the formal state and informal public sphere models can become blurred in practice. The WHA looks like a formal state institution, organised as a voting body of state delegations (many of which are, it must be said, not themselves democratic). However, the WHO lacks the powers of a state, and this resolution in particular consisted of “soft” declarative statements rather than “hard” law.

This case study diverges from the theoretical discussion above in that the relationship between the drug companies and MSF was more antagonistic than my ideal sketch. While GlaxoSmithKline and Pfizer see the matter as satisfactorily resolved, MSF does not regard the matter as settled and continues to push for further concessions.^12^ The sequence of events corresponds better to a picture of managers giving in to outside pressure from campaigners than to a picture of managers welcoming guidance from the public sphere. This is a more realistic expectation. Companies have to apply normative considerations from the public sphere to technical knowledge about internal aspects of the firm, and it may often be easier to do this in dialogue with civil society groups. We should not expect such interactions to meet the classical requirements of consensus-oriented deliberation, and they are very likely to have a more strategic and activist character (Brand et al., 2020; Dawkins, 2021). This does not detract from the case as an example of why something extra is needed to narrow down the options allowed by the MFA, and how engagement with civil society might provide this.

## Democracy-First Business Ethics

Before concluding, it may be helpful to briefly compare my critique of the MFA and my positive proposal with other recent work on the topic. In particular, another set of critics of the MFA have made stronger demands for managers to defer to democracy in interpreting their ethical obligations, a position I refer to as *democracy-first business ethics*.

The point is put most straightforwardly by David Silver (2021). Silver’s argument rests directly on a strong conception of the general authority of democracy: everyone is obliged to obey democratic decisions (within reasonable limits). To this, Silver adds that it is both possible and obligatory to follow the spirit as well as the letter of decisions, and he applies this duty to business managers. Silver’s thesis is substantially similar to Thomas Christiano’s (2010a).

Jeffrey Smith and Ron & Singer reach a similar conclusion via a dialectical engagement with the MFA. Smith (2019) argues that markets should be seen as democratic choices. Democracies regulate markets in order to advance values other than efficiency, and so these values too should figure in managers’ role moralities. Ron and Singer endorse Smith’s point, and add that, in addition to respecting democratic decisions and the “spirit” behind democratic decisions (2020, 151), managers need to be particularly careful to respect the “process” of democratic decision making. This last point lines up with my earlier comments about the need for corporations to refrain from polluting the public sphere if they are to get anything meaningful out of it. As with my comments, this does not necessarily imply any practical departure from to the MFA, which already features a strong prohibition on lobbying (Jaworski, 2013, 2014; Néron, 2016).

What my argument shares with democracy-first business ethics is a demand that managerial decision making should pay more heed to the guidance of democracy. The difference is that democracy-first business ethics follow democratic guidance in general, whereas this paper argues for following democratic guidance in interpreting the demands of the MFA*.* My goal is to advance an internal critique of the MFA and to show how democracy can appear as a solution to a problem of discretion that arises in working out the implications of the MFA. Whether or not managers should defer to democratic demands for things that go against the MFA is not something I take a stance on here.

To elaborate this point, it may be helpful to briefly contrast my argument with a related idea, Abraham Singer’s (2018, 2019) “Justice Failures Approach” (JFA). Singer argues that, just as departures from the ideal of the market increase the ethical obligations on managers, so to do departures from the idea conduct of the state. Insofar as states fail to provide political, social and distributive justice, firms should attempt to fill the gap themselves (cf. Blanc & Al-Amoudi, 2013). Charlie Blunden (2021) distinguishes between two versions of this thesis. On the one hand, the “MFA + ” consists of the MFA plus “market-foreign obligations” of justice which do not run contrary to the MFA. On the other hand, in the full-blown JFA, “market-antithetical obligations” of justice trump efficiency-based obligations in the MFA.

One way of framing my argument is that because the MFA is more uncertain and indeterminate than it appears, expanding to something like the MFA + actually helps to fix an internal problem in the MFA. (This also shows that Blunden’s terminology of “market-foreign” is quite misleading here, since what I have tried to show is that the “ + ” will consist significantly in resolving questions about what kind of market we want to have, not just about other things we could add on top of a market-based morality.) Democracy-first business ethics, by contrast, prioritises “market-antithetical obligations” over the MFA. (In both cases, we view the content of market-foreign and market-antithetical obligations as something to be determined democratically, rather than by an independent moral theory).

Despite the differences, I want to emphasise how my analysis could nonetheless be used to develop the democracy-first position further. I turn to democratising managerial discretion to solve a particular problem with the MFA identified in Sect. 2 of the paper. However, my analysis of how managers can take on board democratic input in Sect. 3 could also be cut loose from this context and used to flesh out democracy-first approaches like those of Silver, Christiano, Smith and Ron & Singer. All of these authors concentrate on the spirit of the law as developed in formal state institutions. If my analysis in Sect. 3 is correct, their approach could benefit from taking more seriously the informal public sphere as a manifestation of democracy from which managers could draw ethical guidance.

This brings me back to PCSR and its critics. The approach to democratising managerial discretion defended here bears some resemblance to the approach of Scherer and Palazzo. Indeed, it is striking that further reflection from Heath’s apolitical and economic starting point has brought us closer to Scherer and Palazzo’s avowedly political view. Nonetheless, there remain important differences. First, whereas PCSR articulates the conditions for a corporation’s legitimacy, my focus is on how a manager should behave. Second, PCSR envisages deliberation on a company’s conduct in general, whereas I have focused on looking to the public sphere to answer the much more limited question of how best to interpret the requirements of the MFA. Related to this point is that PCSR conceives of the company itself as a participant in deliberation, with its own legitimate interests to advance. By contrast, I see the manager as only an audience for this particular deliberation, and not as an advocate for the company.

This last point allows my focus on civil society to avoid some important criticisms of PCSR. Waheed Hussain and Moriarty (2018) emphasise the ways in which the corporation is poorly suited to play a role as an *participant* in deliberation, while Carmen Sabadoz and Singer (2017) emphasise how it is poorly suited as a *forum* for deliberation. Both ask for managers to be a little less self-absorbed and to focus more on the democratic aspects of other parts of civil society, a point with which I very much agree.

## Conclusion

I began by presenting managerial discretion as a general problem for thinking about the role of businesses in society. I then applied this problem in detail to the contemporary theory of business ethics I regarded as strongest, the market failures approach. I showed how this approach allows for much more room for practical disagreement than it initially appears, re-opening the space for problematic managerial discretion. Finally, I sketched how the MFA might draw on democratic input to fill in these gaps, particularly from the informal public sphere.

This amended version of the MFA takes up Jeffrey Smith’s (2019) challenge of steering a delicate balance between an under-politicised, technocratic approach and an over-politicised approach which ignores the competitive nature of business in a market economy. My account opens up space for politics within the MFA, drawing on certain practical elements of PCSR to fill it.

As I have stressed, my focus here has been on how a manager should ideally respond to the problem of discretion. If we want to instead think about the problem from the perspective of the state or citizen groups holding companies to account, this framework can only serve as a starting point. Providing strong incentives to companies requires creating institutions that can set out concrete expectations rather than relying on a looser sense of the will of the people (Hussain & Moriarty, 2014). This directs us towards the model of democratising managerial discretion via institutionalised stakeholder forums. Such institutions may be valuable for pushing companies towards good conduct with regard to the original core tenets of the MFA as well as the democratic supplement I have argued for here.^13^

The politics of lobbying corporations rather than governments is growing in importance (Crouch, 2011). I have argued that this kind of politics is to some extent morally vindicated and should be welcomed. Managers have a duty to listen to civil society campaigns aimed at them, and they should be subject to further criticism (in addition to whatever sparked the original campaigns) when they fail to do so.

## Footnotes

1 The basic idea was set out earlier in (McMahon 1981). However, I focus on Heath’s more detailed and influential articulation.

2 See also (Moon, Crane and Matten 2005). For critique see among others (Hussain and Moriarty 2014; Néron 2010; Sabadoz and Singer 2017; Whelan 2012).

3 Steinberg (2021) provides an alternative example of offsetting market failures (information asymmetries) in the insurance industry.

4 Insofar as it is successful, Carson Young’s (2021) critique of the MFA can also be seen as extending the problem of epistemic uncertainty which I have identified as arising from the problem of the second best. Drawing on Mark Blaug (2007), Young argues the conditions of perfect competition are so stringent they would rule out any attempt to innovate on product quality, and so they cannot be used to help work out which business strategies are compatible with efficiency.

5 An alternative explanation for why some people would be made worse-off by the sugary drinks is that they are simply unaware of the negative health effects (and if they were aware, they would drink less). From the perspective of gains from trade, this is the market failure of *information asymmetry,* or on its own terms this is a breakdown of the mechanism of co-operation Heath calls *information transmission*. If this explanation is preferred, more or less the same argument for a clash with gains from trade could still be made, this time with information asymmetry rather than dynamic preference inconsistency.

6 Of course, this can be difficult to achieve in practice, especially without state support. Such difficulties loom large for iconic multi-stakeholder initiatives such as the Forest Stewardship Council (Moog, Spicer and Böhm 2015).

7 (For a philosophical analysis of this phenomena see Richardson 2003).

8 For attempts to articulate an ideal of the public sphere see, among others (Dryzek 2000; Habermas 1989; Parkinson & Mansbridge 2013).

9 Here I part company with *The Economist*’s worries with which I began; whereas as they see “campaigning groups” as insiders along with “CEOs” and “politicians” in opposition to “the people”, I see such campaigners (and indeed politicians) as part of a democratic system.

10 Apart from anything else, it’s easy to overrate the extent to which the internet has overtaken broadcast media; for example, in a large 2019 survey, 65% of UK respondents and 63% of US respondents reported that their first contact with the day’s news was from an offline source (Newman 2019, 16).

11 Subsidised by wealthy countries and philanthropists such as the Bill & Melinda Gates Foundation, Gavi is today co-leading the COVAX programme to provide Covid-19 vaccinations to poorer countries (www.gavi.org).

12 Today, the focus of activist energy is on the inequitable global distribution of Covid-19 vaccines, with a coalition of civil society organisations campaigning as the “People’s Vaccine Alliance” (https://peoplesvaccine.org/).

13 See (Ciepley 2019) for mechanisms to hold companies to the public interest.
